# Spatial resolution versus data acquisition efficiency in mapping an inhomogeneous system with species diffusion

**DOI:** 10.1038/srep10542

**Published:** 2015-06-02

**Authors:** Fengxiang Chen, Yong Zhang, T. H. Gfroerer, A. N. Finger, M. W. Wanlass

**Affiliations:** 1 University of North Carolina at Charlotte, Charlotte, NC 28223, U.S.A; 2 Wuhan University of Technology, Wuhan, Hubei 430070, China; 3 Davidson College, Davidson, NC 28035, U.S.A; 4 National Renewable Energy Laboratory, Golden, CO 80401, U.S.A

## Abstract

Traditionally, spatially-resolved photoluminescence (PL) has been performed using a point-by-point scan mode with both excitation and detection occurring at the same spatial location. But with the availability of high quality detector arrays like CCDs, an imaging mode has become popular for performing spatially-resolved PL. By illuminating the entire area of interest and collecting the data simultaneously from all spatial locations, the measurement efficiency can be greatly improved. However, this new approach has proceeded under the implicit assumption of comparable spatial resolution. We show here that when carrier diffusion is present, the spatial resolution can actually differ substantially between the two modes, with the less efficient scan mode being far superior. We apply both techniques in investigation of defects in a GaAs epilayer – where isolated singlet and doublet dislocations can be identified. A superposition principle is developed for solving the diffusion equation to extract the intrinsic carrier diffusion length, which can be applied to a system with arbitrarily distributed defects. The understanding derived from this work is significant for a broad range of problems in physics and beyond (for instance biology) – whenever the dynamics of generation, diffusion, and annihilation of species can be probed with either measurement mode.

The performance of electronic and optoelectronic devices depends strongly on the nature and quantity of defects in the constituent materials. Imperfections in the crystal lattice tend to introduce localized states with energy levels lying within the forbidden gap of a semiconductor. When free carriers are injected by an electrical bias or generated by illumination, these levels augment nonradiative recombination by capturing the carriers and providing them with alternative dissipation pathways. This loss competes with desired outcomes, like radiative recombination to produce light in a light-emitting diode or extraction via electronic drift to produce current in a solar cell. More generally, defects lead to two important consequences for optoelectronic device operation: (1) they enhance the recombination of electron-hole pairs which decreases the carrier lifetime; and (2) they increase leakage current which can amplify device noise^1^.

Spatially-resolved optical spectroscopy is widely used for the characterization of individual defects^2^,^3^. One apparent limitation to the spatial resolution is optical diffraction^4^, although resolution beyond the diffraction limit is possible with near-field and other special techniques^2^,^5^,^6^,^7^,^8^,^9^. This work addresses how another effect – carrier diffusion, which exists in virtually all real-world devices – can dramatically impact the spatial resolution under different excitation/detection modes. Carrier diffusion is the mechanism that allows individual microscopic defects to collectively yield a range of important mesoscopic effects. Therefore, on the one hand, understanding the effect of an individual defect is critically important for interpreting mesoscopic behavior; and on the other hand, an individual defect can be used as a sensor for probing mesoscopic phenomena, for instance carrier diffusion, and predicting the “intrinsic” diffusion length in the absence of defects^10^.

Photoluminescence (PL), cathodoluminescence (CL), and laser-beam or electron-beam induced current (LBIC or EBIC) microscopy are powerful techniques for studying carrier transport and recombination in the vicinity of extended defects like dislocations. Generally speaking, according to differences in the manner of excitation and collection, the commonly known spatially-resolved techniques can be divided into three categories: (1) Uniform illumination/Local detection (U/L mode): the sample is illuminated by a large excitation beam and the emission is detected in a spatially-resolved manner, usually via imaging with a CCD camera or mapping point-by-point. In this case, despite the difference in data collection efficiency, the imaging and mapping methods yield about the same spatial resolution. (2) Local excitation/Local detection (L/L mode): by using a confocal optical technique, when the excitation and collection apertures are aligned, the collected signal originates only from the excitation site. PL mapping can be performed in either U/L or L/L mode. (3) Local excitation/Global collection (L/G mode): for example CL, where the electron beam initially produces a highly localized carrier population, but the carriers are free to diffuse before recombining to yield luminescence. Here, a non-confocal method is usually used for signal collection, to include the contribution from carriers that have diffused away from the excitation site. In a diffusion free system, the spatial resolution is determined by the point-spread function of the focused excitation beam, and the spatial resolutions are expected to be more or less the same for all the three modes. However, with diffusion, roughly speaking, the spatial resolution in the U/L and L/G modes is determined by the beam size or diffusion length, whichever is larger. Therefore, for a material with a large diffusion length (i.e. a material of high quality), the spatial resolution is significantly degraded by carrier diffusion. In such a situation, the L/L mode can offer substantially better spatial resolution without changing the beam size or collection optics, based on a simple mathematical consideration that has not been generally appreciated.

The dynamics of carriers in a semiconductor is typically governed by a diffusion equation that contains generation, diffusion, and annihilation (recombination) terms^1^. Similar differential equations are used for many other systems, such as thermal diffusion^11^ and molecular diffusion in biology^12^. To describe the local concentration inhomogeneity centered on an isolated defect (**r** = 0), one normally defines a so-called contrast function
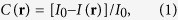
where I_0_ is the signal from the homogeneous area (in our case the defect-free area) and I(**r**) is the signal at position **r**. In the literature, most of the contrast function calculations are performed for CL or EBIC with unique defect geometries, which often results in rather complicated functional forms for C(**r**)^13^,^14^,^15^,^16^. However, the basic component of C(**r**) is always exp(-r/L_d_) with L_d_ being the effective diffusion length, i.e. the carrier depletion near the defect is assumed to decrease exponentially with distance from the defect. This functional form has been used, and indeed nearly taken for granted, in all three of the above mentioned excitation/detection modes^10^,^17^,^18^.

In this report, for the first time, we compare the spatial resolution of the U/L and L/L modes directly, by performing PL mapping on an isolated single defect and on a defect pair in each mode. Our results provide unambiguous experimental proof of the superior spatial resolution achievable with the L/L mode relative to the U/L mode, as supported by our mathematical considerations.

## Results

### A. One-dimensional (1-D) problem

We first consider the L/L mode by starting with a 1-D model. In steady state, the excess carrier density in the semiconductor is governed by the following 1-D continuity equation:
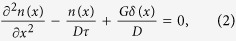
where n(x) represents the carrier density, D is the carrier diffusivity, τ the carrier lifetime or 1/τ the recombination rate, G the generation rate with the assumption that the excitation profile is a delta function, and the diffusion length is defined as 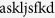. Assuming that the excitation is at x = 0 and an isolated defect at x_0_ > 0 has an infinite recombination rate, we have these boundary conditions: n(x_0_) = 0 and n(±∞) = 0, with x_0_ being the separation between the defect and excitation/detection site. The solution of Eq. (2) satisfying these boundary conditions is:
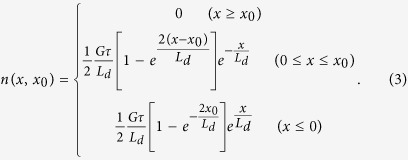


Note that letting x_0_ → ∞ leads to the solution for the defect-free case, i.e. n_0_exp(-|x|/L_d_) with n_0_ = Gτ/(2L_d_). The contrast function can be calculated using the definition C(x_0_) = [n_0_-n(0,x_0_)]/n_0_, which describes the relative reduction of the carrier density at the excitation and detection site x = 0. Explicitly,
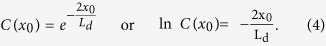


Surprisingly, the slope of - lnC(x_0_) vs. x_0_ is 2/L_d_, a factor of 2 larger than one might initially suspect, which means that the extracted diffusion length would be a factor of 2 smaller than the actual value if assuming C(x_0_) ∝ exp(-x_0_/L_d_).

Although the above 1-D problem has been solved analytically as described above, we introduce an alternative method using a superposition principle to solve the same problem because it will be very useful for the more challenging 2-D case. We note that when the recombination rate at the defect site is much larger than at a general site, the defect can be simulated as an additional negative generation at x_0_ with a generation rate of G_D_ = - G exp(-x_0_/L_d_). The superposition of the two excitations, G at x = 0 and G_D_ at x = x_0_, yields the exact same solution as Eq. (3).

For the U/L mode, the steady-state solution is simply n = gτ in the absence of defects, where g is the generation rate per unit length. Adding a defect is equivalent to introducing a negative generation – Gδ(x), with G = 2gL_d_ at the defect site x = 0. The combined solution is then gτ[1-exp(-|x|/L_d_)], which yields n(0) = 0 and C(x) = exp(-x/L_d_), the well-known form. Clearly, the contrast functions are different for the two excitation/detection modes. The results imply that the effects of a defect should be much more localized in the L/L mode, providing substantially better spatial resolution for resolving nearby defects.

### B. 2-D problem without defect

A 2-D model is appropriate for a relatively thin layer that has nearly uniform carrier density along the perpendicular direction. The excitation site is selected at the origin ***r*** = 0. The diffusion equation in cylindrical coordinates is given as
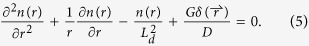
where 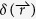 is the 2-D delta function. The solution of Eq. (5) is a modified Bessel function K_0_(ξ) with ξ = r/L_d_. If we apply the boundary conditions n(∞) = 0 and 
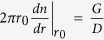
 with r_0_ → 0, the solution will be
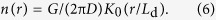


Though K_0_(ξ) diverges at ξ = 0, ξK_0_(ξ) is integrable near ξ = 0. We can replace K_0_(0) with the average of K_0_(ξ) over a small circle of radius ε:^19^
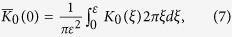
which is equivalent to the effect of having a finite experimental probe. For example, in the PL measurement ε could be related to the diffraction limit spot size.

### C. Superposition principle in 2-D with defect

**Figure 1:**
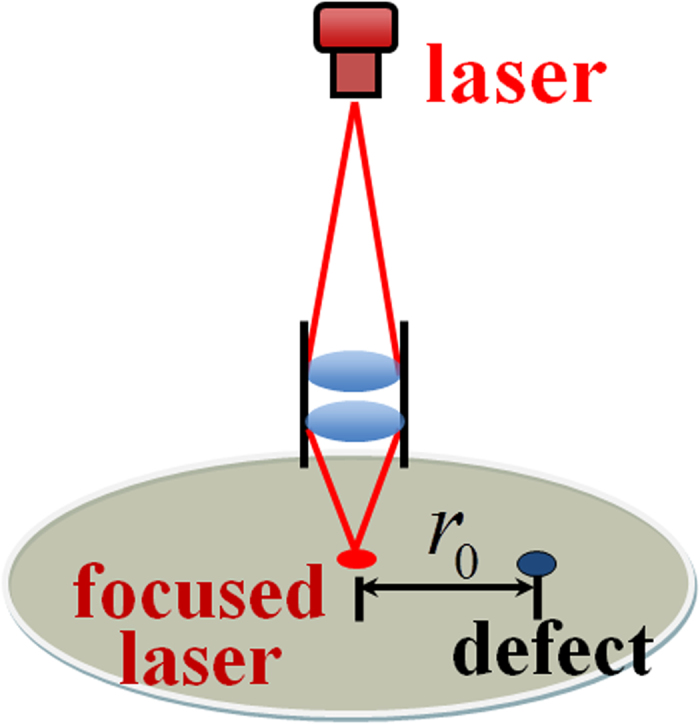
Schematic illustration of PL mapping in the L/L mode.

Now we use the superposition principle to derive the carrier distribution in the vicinity of a defect. First the L/L mode is considered with the configuration shown schematically in Fig. 1.

Suppose that, in an infinitely large plane without defects, the carrier density generated by a laser beam at r = 0 is *n*_*0*_. At a distance *r*_*0*_ away, the density will reduce to *ηn*_*0*_, where *η* is a decay function that depends on the distance *r*_*0*_but is independent of the density at r = 0, varying in the range of *0* ≤ *η* ≤ *1*. If a defect with an infinite recombination rate is placed at *r*_*0*_, a negative generation source yielding a density *–ηn*_*0*_ is needed to ensure that the density at the defect site remains zero. Now using the superposition principle, the net carrier density at r = 0 is the superposition of the contributions from the incident light at r = 0 and the negative generation source at *r*_*0*_, which totals *n*_*0*_*-η*^*2*^*n*_*0*_.

According to the definition of the contrast function, we have
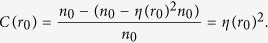


Thus we can conclude that the contrast function *C* in the 2-D case is also the square of the decay function *η*. Applying the analytic solution from Part (B), the decay function *η* is equal to 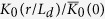, and
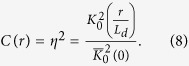


We note that although 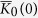 is finite, its magnitude depends on the specific value of ε used in Eq. (7). In principle, an appropriate value of ε can be selected based on the excitation beam size to give a meaningful value of 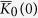. In practice, this is rather inconvenient and also not necessary. Because the technique is most useful when the excitation beam size is significantly smaller than the diffusion length, skipping the r = 0 point will not have any major impact on the accuracy of the derived diffusion length, and 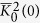 in Eq. (8) can be treated as a pre-factor in the fitting.

For the U/L mode, the steady-state solution is similar to that in the 1-D case. We assume n = gτ in the absence of defects. Near the defect at r_0_ = 0, the effect of the defect can be described by a negative generation as -G_D_/(2πD)K_0_(r/L_d_), where -G_D_ represents the effective generation rate of the defect. As in the 1-D case, a defect is assumed to have an infinite recombination rate at the defect site and zero lateral extension, so we have
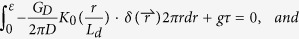

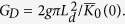


Then the carrier density is 
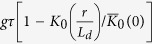
20, and the contrast function is
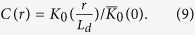


Again in fitting the experimental data, the r = 0 point can be skipped and 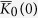 can be treated as a pre-factor.

We note that these results are similar to what we obtained in the 1-D case, where the contrast function is η = exp(-x_0_/L_d_) for the U/L mode and η^2^ = exp(–2x_0_/L_d_) for the L/L mode. Therefore, in an ideal situation, if two defects separated by δx_0_ are resolvable by a detection system operated in the U/L mode, then they can be resolved equally well when the separation is reduced to δx_0_/2 by the same detection system operated in the L/L mode.

### D. Experimental comparison of the two excitation/detection modes

**Figure 2:**
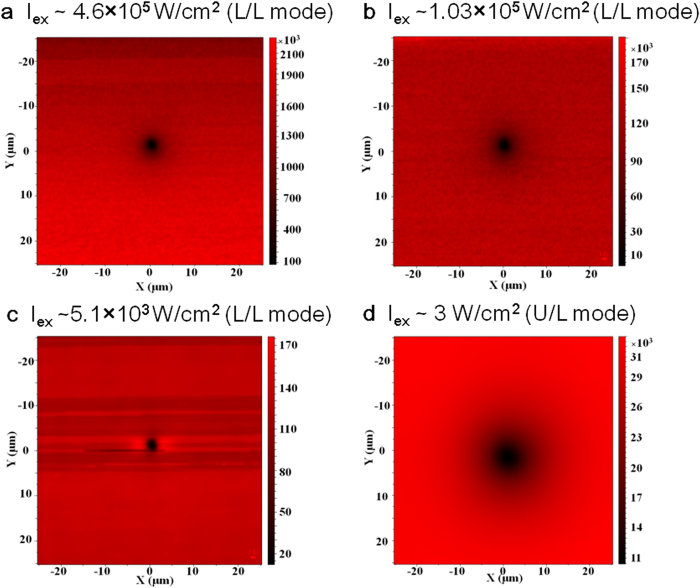
PL maps of a single dislocation in GaAs measured under two excitation/detection modes: (**a**–**c**) for the L/L mode, and (**d**) for the U/L mode. The vertical bars indicate the relative intensities in photon counts.

**Figure 3:**
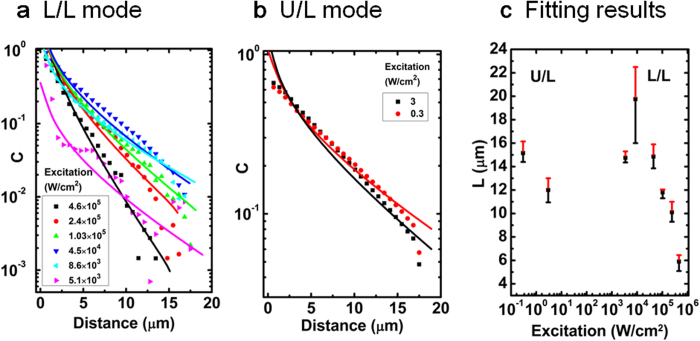
Circularly averaged radial contrast functions deduced from the PL mapping data, shown as symbols in (**a**) for the L/L mode and (**b**) for the U/L mode. Solid lines are theoretical fits using Eq. (8) and Eq. (9), respectively, for the L/L and U/L modes. (**c**) Fitting results of diffusion lengths for the L/L mode and U/L mode on a single defect. The points represent the best fits, the error bars give the variation ranges that are able to offer reasonably good fits.

We have shown previously, using PL mapping with either the L/L or U/L mode^10^,^21^, that the effect of one isolated extended defect (e.g., a dislocation) manifests as a circular dark region with emission intensity that increases with distance from the defect, asymptotically approaching that of the defect free region. Here we first illustrate how the impact range of the same isolated defect may appear very different under the two modes, as shown in Fig. 2a–2c for the three excitation densities under the L/L mode, and Fig. 2d for one example of the U/L mode. Figure 3a–3b depict the circularly averaged radial contrast functions computed using Eq. (1) for all of the data taken under the two different modes. Although within the L/L mode the size of the dark region varies visibly with the excitation density as a result of the competition between point defects (un-resolvable in this type of measurement) and the extended defect^10^, the impact range of the defect under the U/L mode is clearly much larger than what is observed in the L/L mode. If one simply modeled the data using the common contrast function C(r) ~ exp(-r/L), one would deduce an effective diffusion length from the L/L data that is approximately half the actual diffusion length obtained from the U/L data. However, when the contrast functions defined in Eq. (8) and Eq. (9) are used for the L/L and U/L modes respectively, the diffusion lengths are generally found to be consistent across all measurements, accurately reflecting the intrinsic material property of the same region. The fitting results for the diffusion length are shown in Fig. 3c. The variation within each mode is due to a range of other subtle effects, which have been discussed elsewhere^10^,^21^. The results suggest that the carrier diffusion length can vary approximately from 6 to 20 μm in this particular region of the sample with varying excitation density. These values are typical for high quality GaAs epilayers^22^. More importantly, the faster decay of the contrast function under the L/L mode suggests that substantially better spatial resolution can be achieved in the L/L mode when compared with the U/L mode.

**Figure 4:**
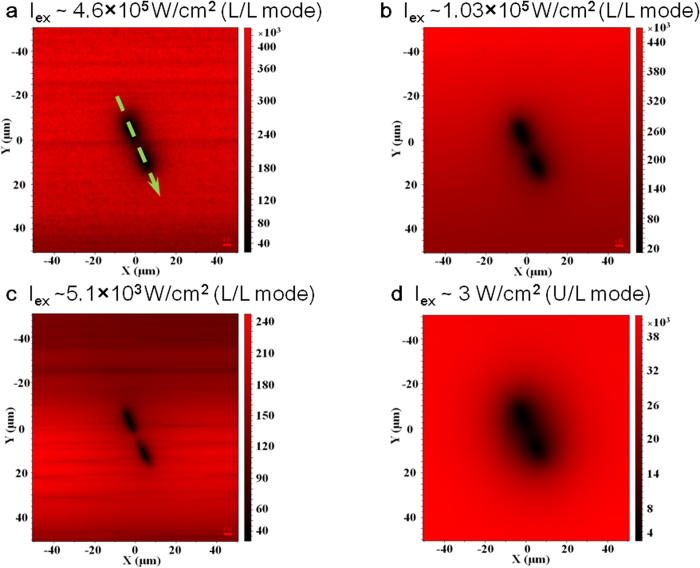
PL maps of a dislocation pair in GaAs measured under two excitation/detection modes: (**a**–**c**) for the L/L mode and (**d**) for the U/L mode. The vertical bars indicate the relative intensities in photon counts.

**Figure 5:**
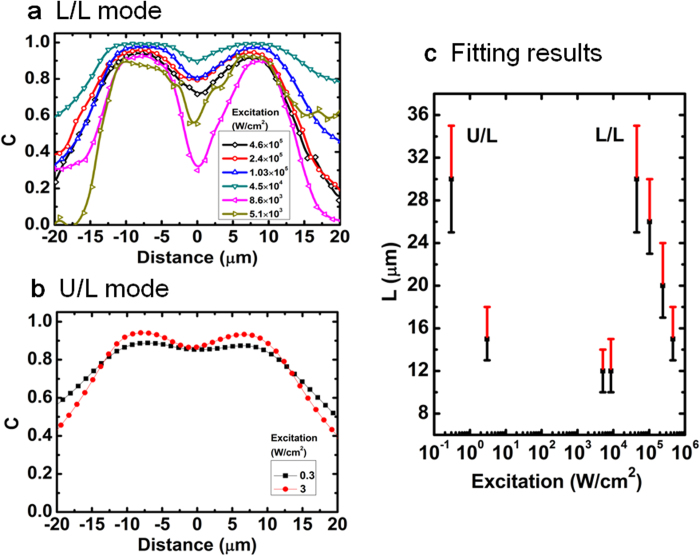
Contrast functions computed from the PL mapping data of the doubl*et al*ong the line of symmetry passing through the two defects (**a**) for the L/L mode and (**b**) for the U/L mode. The solid lines are guides to the eye. (**c**) Diffusion lengths derived from fits to the L/L mode and U/L mode contrast profiles for the defect pair.

Next we demonstrate that the L/L mode can indeed yield much better spatial resolution when attempting to resolve nearby defects. For this purpose, we use a pair of dislocations separated by a distance (~ 15 μm) that is slightly larger than or about one half of the diffusion length, depending on the excitation condition. Selected PL mapping results for the doublet are shown in Fig. 4 for the two modes, with the corresponding contrast functions given in Fig. 5. The doublet is clearly resolved in the L/L mode, as shown in the PL maps Fig. 4a–4c and contrast functions Fig. 5a. The resolution is particularly good at low excitation intensity where the diminished carrier density leads to a reduction in the diffusion length^10^. For comparison, the doublet is barely resolved in the U/L mode PL map shown in Fig. 4d. This shortcoming is also evident in the contrast functions for the U/L mode data shown in Fig. 5b. Except for the loss of circular symmetry, the results for the doublet are qualitatively similar to those of the singlet. For instance, the influence range of the defect varies with excitation density in a similar fashion for the L/L mode. The contrast functions shown in Fig. 5a–5b are slightly asymmetric, which might be due to a real material difference, but since the asymmetry is more apparent for the weaker signal, it may also be attributed to noise. We have performed the same fitting analysis that was used for the singlet on each side of the doublet, yielding averaged diffusion lengths shown in Fig. 5c. The trends are qualitatively the same as those shown in Fig. 3c, except that the deduced diffusion lengths are somewhat larger overall. This variation is understandable because the recombination dynamics can depend sensitively on the density of microscopic defects that may vary across a large wafer^10^. Quantitatively, the ratio γ = C(r_d_)/C(0) can be used to measure the improvement in resolution, where C(r_d_) and C(0) are respectively the contrast function values at the defect sites (averaged) and the center of the line connecting them. For data taken in the two different modes but having comparable diffusion lengths L_d_ ≈ 12–13 μm (e.g., Fig. 4c and Fig. 4d), we get γ_L/L_ ≈ 1.64–3.05, and γ_U/L_ ≈ 1.08. Altogether, the results unambiguously confirm the prediction that the L/L mode can offer vastly superior spatial resolution when the mechanism of diffusion is important.

## Discussion

We have demonstrated both theoretically and experimentally that two generation/detection modes – (local generation)/(local detection) and (uniform generation)/(local detection) – can yield very different spatial resolution when species diffusion is present. The first mode, which is referred to as L/L mode, is shown to have far superior spatial resolution compared to the second (U/L) mode. The improvement in spatial resolution, approximately a factor of 2, can be attributed to a steeper contrast function, approximately exp(-2x/L_d_) vs. exp(-x/L_d_), when the nominal diffusion length is L_d_. The theoretical prediction has been proved unambiguously with PL mapping data and analysis on single and doublet defects in GaAs. We conclude that, even though the U/L mode has superior collection efficiency, it comes at the expense of reduced spatial resolution when species diffusion is significant. In light of the fact that both modes are widely used for studying spatial inhomogeneity and diffusion in a wide variety of natural systems (e.g., electrons in a semiconductor, thermal transport in a construction material, or molecular diffusion in a biological cell), the principle derived in this work should garner broad interest and impact scientific disciplines well beyond the field of semiconductors.

## Methods

We used a quasi-two dimensional system – namely, a double heterostructure of GaInP/GaAs/GaInP, in which the photo-generated carriers are confined within the thin GaAs layer between GaInP barriers such that the GaAs interfaces are passivated to eliminate interface recombination^10^. The sample was grown by metal-organic vapor phase epitaxy (MOVPE) on a semi-insulating GaAs substrate, and has a very low as-grown threading dislocation density on the order of 10^3^ cm^−2^. All experiments were conducted at room temperature on a Horiba LabRAM HR800 confocal Raman microscope. For the L/L mode, a 633 nm laser was used with a 100x microscope lens (NA = 0.9) that can produce an excitation spot size with a diameter of about 860 nm. The PL signal was focused to the confocal aperture, dispersed by a spectrometer, and detected by a CCD array. For the U/L mode, we used an 808 nm laser coupled through the white-light illumination port of the microscope with a Kohler optics system that can generate uniform illumination under the same microscope lens. The PL signal was collected in two ways: (1) mapping as in the L/L mode or (2) imaging using the microscope camera. The two collection methods yielded more or less the same results, although the signal-to-noise ratio of the latter method, in line with the commonly adopted more efficient way of taking data^3^,^21^, was not as good. A better camera would presumably alleviate this deficiency. We used the same data collection method for comparing the L/L and U/L modes to ensure that the only difference between the experiments was the excitation beam shape (i.e., δ-function-like vs. uniform).

## Additional Information

**How to cite this article**: Chen, F. *et al*. Spatial resolution versus data acquisition efficiency in mapping an inhomogeneous system with species diffusion. *Sci. Rep.* **5**, 10542; doi: 10.1038/srep10542 (2015).
